# Future think: cautiously optimistic about brain augmentation using tissue engineering and machine interface

**DOI:** 10.3389/fnsys.2015.00072

**Published:** 2015-05-19

**Authors:** E. Paul Zehr

**Affiliations:** ^1^Rehabilitation Neuroscience Laboratory, University VictoriaVictoria, BC, Canada; ^2^Human Discovery Science, International Collaboration on Repair DiscoveriesVancouver, BC, Canada; ^3^Centre for Biomedical Research, University of VictoriaVictoria, BC, Canada; ^4^Division of Medical Sciences, University of VictoriaBC, Canada; ^5^School of Exercise Science, Physical, and Health Education, University of VictoriaVictoria, BC, Canada; ^6^Zanshin Consulting, Inc.Victoria, BC, Canada

**Keywords:** rehabilitation, enhancement, transhumanism, stem cell, engraftment, brain machine, neuroprosthetics, Asimov

“There is no sensible way in which we must take the possibility of misuse into account before determining that something is an enhancement.” —John Harris in “Enhancing Evolution” (Harris, 2007)

*“I need to wire the armor directly into my brain. Extremis could do that… I need to be the suit… I need to grow new connections”* —Tony Stark talking about integration with the Iron Man armor in the graphic novel “Extremis” by Warren Ellis (Ellis, 2007)

## Introduction

Augmentation of brain function can imply restoration of function lost due to pathology or injury. On the other hand, techniques, approaches, and technologies used for brain augmentation in restoration can also amplify the range of human abilities in those without pathology. For example, non-invasive brain stimulation using transcranial magnetic stimulation (TMS) was originally applied for investigative and diagnostic purposes in neurological injury (Nollet et al., 2003). While TMS continues to be used in brain mapping and restoration of functional output (Romero et al., 2011; Bestmann and Krakauer, 2015), recent clinical applications in otherwise “healthy people” are widening. These include enhancing attention and vigilance (Nelson et al., 2014), motor learning (Cantarero et al., 2015) and various methods to “… improve attention, perception, memory and other forms of cognition…” (Clark and Parasuraman, 2014).

Technological augmentation of so-called “normal” human function moves us away from the functional limitations of our species and closer to “super” human function (Zehr, 2015), as with suggestions found in the transhumanist literature (Mcnamee and Edwards, 2006). Future applications of emerging technology can continue to shift us from our subspecies of *homo sapiens sapiens* to the transformative *homo sapiens technologicus*—a species that uses, fuses and integrates technology to enhance its own function (Zehr, 2011, 2015).

While there are many related approaches, this opinion article explores brain augmentation using approaches delimited to internally implanted biological enhancements (e.g. tissue engineering), and internal/external technological hardware [e.g., brain machine interface (BMI)].

## Tissue engineering and stem cell chimeras

The pace of discoveries in the field of tissue engineering in regenerative medicine applications continues to accelerate (Leach et al., 2010; Elliott Donaghue et al., 2014). In 2013, Xiaoning Han and colleagues in the laboratories of Steven Goldman and Maiken Nedergaard at the University of Rochester Medical Center published a paper examining the possibility of augmenting neural processing ability of one species by surgically transplanting cells from the brain of another “more advanced” species (Han et al., 2013).

This research team was concerned with “biocompatability” in the mouse brain with certain evolutionary adaptations in human astroglial cells which are much larger with more complex structure than those found in the mouse. These astrocytes, which don't produce electric signals like neurons, are considered critical as physiological support and protection for the processing neurons, particularly in calcium signaling. This signaling is crucial for overall brain activity and human astrocytes operate threefold faster than those found in the mouse.

This generated the question: what would happen if you grafted human glial progenitor cells—the stem cells in the brain that would normally become astrocytes—into the forebrain of immunosuppressed mice? For how long would human cells survive in the mouse brain, and, critically, would the human astrocytes offer any behavioral advantage to the mice hosting the implanted cells?

Han and colleagues discovered that, in the mouse hippocampus, human glial cells thrived, and propagated calcium signals at the rate usually found in the human brain. Moreover, there was strengthened signal transmission between neurons (that is, long-term potentiation underlying learning and memory formation).

Critically, the functioning transplanted stem cells augmented behavior in the mice, including maze learning, fear conditioning, and enhanced ability to identify and find new objects in the murine habitat. The augmented chimeric mice with engrafted human glial cells essentially had improved all-around performance and this study represented an important test that cross-species grafting techniques could be a useful way to modify and augment (and examine in pathology) brain function.

Later, Martha Windrem and colleagues from the same laboratories, used an expanded protocol to examine the long term effects of engrafting human glial progenitor cells into the forebrains of neonatal mice (Windrem et al., 2014). Quite dramatically, there was a steady fall in murine cells coincident with an in increase in human cell content in the mouse brain. This proportional shift was so strong that after 1 year the glial progenitor cells found in the mouse forebrain populations were almost entirely of human origin.

That implanted human cells “outcompeted” and eventually replaced and “infected” the initial host mouse cells was an unexpected outcome. Windrem et al. “were surprised to note that the forebrains of these animals were often composed primarily of human glia and their progenitors” (Windrem et al., 2014).

## External technological hardware and augmentation with brain machine interface

The interface of physiology and engineering represented by brain computer or brain machine interface has a history of success in non-human and human applications for restoration of function (Lebedev and Nicolelis, 2006; Wang et al., 2010; Shih et al., 2012). This tremendous success raises new questions. For example, is it possible to attach a biological brain to an external circuit and establish a functional connection for learning? This question was addressed by Theodore Berger and colleagues who showed that an input/output (stimulation and recording) neuroprosthetic interface enhanced memory function subserved by the rodent hippocampus and could overcome memory deficits mimicking natural damage (Berger et al., 2011). This approach was extended to restoration of cognitive decision making using a neuroprosthetic interfaced into the prefrontal cortex in the rhesus monkey (Hampson et al., 2012).

Simeon Bamford and colleagues established a proof of principle for direct applications of brain-machine neuroprosthetics in motor learning using cerebellar motor control and learning circuitry as the model (Prueckl et al., 2011; Bamford et al., 2012). They envisaged engineering scenarios that could meet the challenge required for truly integrated neuroprosthetics—a closed loop system. Such a system would be able to send to and receive from the brain inputs and processing to control devices that supplement the functionality of the brain itself (Bamford et al., 2012).

Cerebellar circuits are essential to classical conditioning of the eye-blink reflex (Cheron et al., 2013). In this protocol a conditioned stimulus (CS; a neutral stimulus like a sound) is paired with an unconditioned stimulus (US; e.g., an airpuff to the eye to evoke a blink reflex).

Initially only the US causes the blink reflex but over time the CS can evoke the blink in the absence of the US. Bamford and colleagues used the known input and output properties of the cerebellum to guide the development of a prototype chip fabricated as a microcircuit. Real data from anaestethized animals were used calibration and training. The circuit could be “conditioned in a manner very similar to that of a real intact cerebellum” thus establishing partial brain function replication using BMI (Bamford et al., 2012).

Herreros and colleagues successfully connected to the brain of an anesthetized rat as a “step toward the development of neuro-prostheses that could recover lost learning functions in animals and, in the longer term, humans” (Herreros et al., 2014). Most recently, this approach was used effectively in interfacing with the rodent brain for testing closed loop motor learning in real time (Hogri et al., 2015). These data lay the groundwork for refining future neuroprosthetics as well as creating a useful system for testing motor learning theories.

Miguel Pais-Vieira and colleagues, part of the Nicolelis and Lebedev groups, demonstrated that brain-machine interface concepts could be extended to brain-to-brain interfaces for shared information processing (Pais-Vieira et al., 2013). In this compelling research, two rats had electrode arrays implanted into the sensorimotor areas. One rat served as an “encoder” of sensorimotor information during performance of either a tactile or visual task. The cortical activity generated in the brain of the “encoder rat” was monitored and then relayed to a second “receiving” or “decoder” rat, located in a distant laboratory.

The brain of the “decoder rat” was electrically stimulated through the implanted electrode array based on the timing and pattern of activity received from the “encoder rat.” The behavior of the “decoder rat” was directed by this activity and subsequently made similar task choices as did the “encoder rat.” Thus, the distant “decoder rat” was taught by the neural traffic generated by the initial activity of the “encoder rat” and relayed by the direct brain-to-brain coupling afforded in this novel “artificial communication channel” (Pais-Vieira et al., 2013). This shows that rats linked through brain-to-brain electrode arrays could learn complex, cooperative, goal-directed behaviors.

A related human test of brain-to-brain interaction was conducted by Grau et al. (2014). They used non-invasive methodologies of electroencephalography (EEG) for signal detection at the “source” brain (essentially the “encoder” rat above) and TMS for transmission to the “receiver” brain (the “decoder” rat analog of Pias-Vieira et al.) to establish that direct communication between the brains of conscious humans was possible. This study focused only on transmission of simple language but heralds the future arrival of more complex communication.

## Conclusion—optimism balanced with some cautious forethought

Stem cell technologies continue to show promise and have the most imminent restorative applications in neurodegenerative disorders such as Alzheimer's disease, Parkinson's disease, and stroke. Critically, tissue engineering may help bootstrap this field while techniques for parallel non-invasive brain monitoring develop. While considerable progress continues, immune response and acute inflammation with implantations and microelectrode insertions into the brain present limitations on long-term viability of some approaches (Richter et al., 2011; Fernandez et al., 2014; Groothuis et al., 2014).

Applications of stem cell technologies in augmenting the “normal” range of human brain function await discovery. Possible avenues discoveries of superior cell function in specialized systems in other animals that may see implantation in humans to augment functions unrelated to processing speed. Assuming improvements in achieving robust and behaviorally-relevant interfaces allowing facile access to discrete input and output pathways, proven, and nascent BMI methodologies have implications for restoration of brain function on a large scale. As the dovetailing of stem cell technology and brain machine interface continues, I suggest we must also pay attention to the issues of security and ethics.

As for security, connecting a machine so the human operator can access the functional capacity of the machine also allows the machine access to the functional capacity of the human. In 1942 the scientist and science fiction writer Isaac Asimov presaged these concerns when describing artificial intelligence and robotics.

In his influential 1942 short story “Runaround,” (later found in the book “I, Robot” (Asimov, 1950) Asimov laid down his “Three Laws of Robotics” which aimed to protect the sanctity of human life. However, such concerns may be rendered moot if future neural interfaces function indistinguishably from the user since it enacts part of the control system that manifests as the will of the user. This means it may actually be impossible to separate between the actions of the neural interface and that of its wearer.

Assuming such convergence, this places a higher order of responsibility on such “augmented” users. I've reworked Asimov's Three Laws below to apply to the complexities of machine-brain-machine interfaces.

An augmented user with a neural interface:

- may not injure a human being or, through inaction, allow a human being to come to harm (Law 1);
- must protect its own existence as long as such protection does not conflict with Law 1 (Law 2).

In fact, this yields Two Laws, since Asimov's declaration (original Law 2) that a robot “must obey order given to it by human beings” is irrelevant. In this future look, the user is the interface and the term human being applies to all and related subspecies (e.g., *homo sapiens sapiens* and *homo sapiens technologicus*; Zehr, 2015).

As for ethical considerations, this sets the stage for complete fusion between trans-species biology and neuroengineering. This will bring us to real life artificial-human brain hybrids and increased applications to enhance and augment innate function rather than simply recover lost function. This includes the extension of the concept of brain augmentation to include the “global brain” suggested by Kyriazis (2015). Future applications to augmentation of otherwise healthy and intact brain function may well be in “the new wave of human enhancement” (Harris, 2007).

Yet, the issue of brain augmentation should proceed with appropriate caution in neurologically intact “normally functioning” people. The comment of Rudolf Jaenisch—in the context of the human gene editing controversy (Baltimore et al., 2015; Cyranoski, 2015; Vogel, 2015)—that “We need some principled agreement that we want to enhance humans in this way or we don't” (Wade, 2015) has resonance here.

Using the examples of brain augmentation discussed above, what other intact cellular interactions in the brain are disrupted by the effect of transpecies implants? What changes in brain structure and function may arise from long-term neuroprosthetic interface? What are the implications for what we now accept as “normal human behavior” and functional capacity?

Especially we need to establish what societal boundaries—if any—we will place on multi-species transplants and what does this mean for the concept of species itself? Many of the related ethical and moral issues are addressed elsewhere in more detail (Clark, 2014; Clark and Parasuraman, 2014; Kennedy, 2014; Hildt, 2015). Along the way forward it remains for us as scientists, engineers, and future users of brain augmentation methodologies to proceed with conviction and purpose, but also with suitable care and caution. Establishing the context for conviction, care and caution must also include dialogue with all members of our society including the general lay public.

### Conflict of interest statement

The author declares that the research was conducted in the absence of any commercial or financial relationships that could be construed as a potential conflict of interest.

## References

[B1] Asimov I. (1950). I, Robot. New York, NY: DoubleDay

[B2] Baltimore B. D. Berg P. Botchan M. Carroll D. Charo R. A. Church G. (2015). A prudent path forward for genomic engineering and germline gene modification. Science 348, 36–38 10.1126/science.aab1028 25791083 PMC4394183

[B3] Bamford S. A. Hogri R. Giovannucci A. Taub A. H. Herreros I. Verschure P. F. M. J.. (2012). A VLSI field-programmable mixed-signal array to perform neural signal processing and neural modeling in a prosthetic system. IEEE Trans. Neural Syst. Rehabil. Eng. 20, 455–467. 10.1109/TNSRE.2012.2187933 22481832

[B4] Berger T., W. Robert E. H. Dong S. Anushka G. Vasilis Z. M. Sam A. D. (2011). A cortical neural prosthesis for restoring and enhancing memory. J. Neural Eng. 8, 046017. 10.1088/1741-2560/8/4/046017 21677369 PMC3141091

[B5] Bestmann S. Krakauer J. (2015). The uses and interpretations of the motor-evoked potential for understanding behaviour. Exp. Brain Res. 233, 679–689. 10.1007/s00221-014-4183-7 25563496

[B6] Cantarero G. Spampinato D. Reis J. Ajagbe L. Thompson T. Kulkarni K.. (2015). Cerebellar direct current stimulation enhances on-line motor skill acquisition through an effect on accuracy. J. Neurosci. 35, 3285–3290. 10.1523/JNEUROSCI.2885-14.2015 25698763 PMC4331640

[B7] Cheron G. Dan B. Márquez-Ruiz J. (2013). Translational approach to behavioral learning: lessons from cerebellar plasticity. Neural Plast. 2013:853654. 10.1155/2013/853654 24319600 PMC3844268

[B8] Clark V. P. (2014). The ethical, moral and pragmatic rationale for brain augmentation. Front. Syst. Neurosci. 8:130. 10.3389/fnsys.2014.00130 25100951 PMC4105582

[B9] Clark V. P. Parasuraman R. (2014). Neuroenhancement: enhancing brain and mind in health and in disease. Neuroimage 85, 889–894. 10.1016/j.neuroimage.2013.08.071 24036352

[B10] Cyranoski D. (2015). Ethics of embryo editing divides scientists. Nature (London) 519, 272–272. 10.1038/519272a 25788074

[B11] Elliott Donaghue I. Tam R. Sefton M. V. Shoichet M. S. (2014). Cell and biomolecule delivery for tissue repair and regeneration in the central nervous system. J. Control. Release 190, 219–227. 10.1016/j.jconrel.2014.05.040 24878181

[B12] Ellis W. (2007). Invincible Iron Man: Extremis. New York, NY: Marvel Comics.

[B13] Fernandez E. Greger B. House P. A. Aranda I. Botella C. Albisua J.. (2014). Acute human brain responses to intracortical microelectrode arrays: challenges and future prospects. Front. Neuroeng. 7:24. 10.3389/fneng.2014.00024 25100989 PMC4104831

[B14] Grau C. Ginhoux R. Riera A. Nguyen T. L. Chauvat H. Berg M.. (2014). Conscious brain-to-brain communication in humans using non-invasive technologies. PLoS ONE 9:e105225. 10.1371/journal.pone.0105225 25137064 PMC4138179

[B15] Groothuis J. Ramsey N. F. Ramakers G. M. J. Van Der Plasse G. (2014). Physiological challenges for intracortical electrodes. Brain Stimulation 7, 1–6. 10.1016/j.brs.2013.07.001 23941984

[B16] Hampson R. E. Greg A. G. Vasilis M. Dong S. Ioan O. Lucas S.. (2012). Facilitation and restoration of cognitive function in primate prefrontal cortex by a neuroprosthesis that utilizes minicolumn-specific neural firing. J. Neural Eng. 9:056012. 10.1088/1741-2560/9/5/056012 22976769 PMC3505670

[B17] Han X. Chen M. Wang F. Windrem M. Wang S. Shanz S.. (2013). Forebrain engraftment by human glial progenitor cells enhances synaptic plasticity and learning in adult mice. Cell Stem Cell 12, 342–353. 10.1016/j.stem.2012.12.015 23472873 PMC3700554

[B18] Harris J. (2007). Enhancing Evolution: the Ethical Case for Making Better People. Princeton, NJ: Princeton University Press.

[B19] Herreros I. Giovannucci A. Taub A. H. Hogri R. Magal A. Bamford S. (2014). A cerebellar neuroprosthetic system: computational architecture and *in vivo* test. Front. Bioeng. Biotechnol. 2:14 10.3389/fbioe.2014.00014 PMC4126458 25152887

[B20] Hildt E. (2015). What will this do to me and my brain? Ethical issues in brain-to-brain interfacing. Front. Syst. Neurosci. 9:17. 10.3389/fnsys.2015.00017 25762903 PMC4340163

[B21] Hogri R. Bamford S. A. Taub A. H. Magal A. Giudice P. D. Mintz M. (2015). A neuro-inspired model-based closed-loop neuroprosthesis for the substitution of a cerebellar learning function in anesthetized rats. Sci. Rep. 5:8451. 10.1038/srep08451 25677559 PMC4327125

[B22] Kennedy P. (2014). Brain-machine interfaces as a challenge to the ‘moment of singularity’. Front. Syst. Neurosci. 8:213. 10.3389/fnsys.2014.00213 25565984 PMC4269110

[B23] Kyriazis M. (2015). Systems neuroscience in focus: from the human brain to the global brain? Front. Syst. Neurosci. 9:7. 10.3389/fnsys.2015.00007 25705180 PMC4319389

[B24] Leach J. B. Achyuta A. K. H. Murthy S. K. (2010). Bridging the divide between neuroprosthetic design, tissue engineering and neurobiology. Front. Neuroeng. 2:18. 10.3389/neuro.16.018.2009 20161810 PMC2821180

[B25] Lebedev M. A. Nicolelis M. A. L. (2006). Brain-machine interfaces: past, present and future. Trends Neurosci. 29, 536–546. 10.1016/j.tins.2006.07.004 16859758

[B26] Mcnamee M. J. Edwards S. D. (2006). Transhumanism, medical technology and slippery slopes. J. Med. Ethics 32, 513–518. 10.1136/jme.2005.013789 16943331 PMC2563415

[B27] Nelson J. T. Mckinley R. A. Golob E. J. Warm J. S. Parasuraman R. (2014). Enhancing vigilance in operators with prefrontal cortex transcranial direct current stimulation (tDCS). Neuroimage 85, Pt 3, 909–917. 10.1016/j.neuroimage.2012.11.061 23235272

[B28] Nollet H. Van Ham L. Deprez P. Vanderstraeten G. (2003). Transcranial magnetic stimulation: review of the technique, basic principles and applications. Vet. J. 166, 28–42. 10.1016/S1090-0233(03)00025-X 12788015

[B29] Pais-Vieira M. Lebedev M. Kunicki C. Wang J. Nicolelis M. A. L. (2013). A brain-to-brain interface for real-time sharing of sensorimotor information. Sci. Rep. 3:1319. 10.1038/srep01319 23448946 PMC3584574

[B30] Prueckl R. Taub A. H. Herreros I. Hogri R. Magal A. Bamford S. A. (2011). Behavioral rehabilitation of the eye closure reflex in senescent rats using a real-time biosignal acquisition system, in Engineering in Medicine and Biology Society, EMBC, 2011 Annual International Conference of the IEEE. (Boston, MA). 10.1109/IEMBS.2011.6091045 22255268

[B31] Richter A. Kruse C. Moser A. Hofmann U. G. Danner S. (2011). Cellular modulation of polymeric device surfaces: promise of adult stem cells for neuroprosthetics. Front. Neurosci. 5:114. 10.3389/fnins.2011.00114 22013407 PMC3189638

[B32] Romero J. R. Ramirez D. M. Aglio L. S. Gugino L. D. (2011). Brain mapping using transcranial magnetic stimulation. Neurosurg. Clin. N.Am. 22, 141–152. 10.1016/j.nec.2010.11.002 21435567

[B33] Shih J. J. Krusienski D. J. Wolpaw J. R. (2012). Brain-computer interfaces in medicine. Mayo Clin. Proc. 87, 268–279. 10.1016/j.mayocp.2011.12.008 22325364 PMC3497935

[B34] Vogel G. (2015). Embryo engineering alarm. Science 347:1301. 10.1126/science.347.6228.1301 25792311

[B35] Wade N. (2015, March 19). Scientists seek ban on method of editing the human genome. New York Times.

[B36] Wang W. Collinger J. L. Perez M. A. Tyler-Kabara E. C. Cohen L. G. Birbaumer N.. (2010). Neural interface technology for rehabilitation: exploiting and promoting neuroplasticity. Phys. Med. Rehabil. Clin. N.Am. 21, 157–178. 10.1016/j.pmr.2009.07.003 19951784 PMC2788507

[B37] Windrem M. S. Schanz S. J. Morrow C. Munir J. Chandler-Militello D. Wang S.. (2014). A competitive advantage by neonatally engrafted human glial progenitors yields mice whose brains are chimeric for human glia. J. Neurosci. 34, 16153–16161. 10.1523/JNEUROSCI.1510-14.2014 25429155 PMC4244478

[B38] Zehr E. P. (2011). Inventing Iron Man: the Possibility of a Human Machine. Baltimore, MD: Johns Hopkins University Press.

[B39] Zehr E. P. (2015). The potential transformation of our species by neural enhancement. J. Mot. Behav. 47, 73–78. 10.1080/00222895.2014.916652 25575224 PMC4299867

